# Chronic Cadmium Exposure Stimulates SDF-1 Expression in an ERα Dependent Manner

**DOI:** 10.1371/journal.pone.0072639

**Published:** 2013-08-28

**Authors:** Esmeralda Ponce, Natalie B. Aquino, Maggie C. Louie

**Affiliations:** Department of Natural Sciences and Mathematics, Dominican University of California, San Rafael, California, United States of America; National Cancer Center, Japan

## Abstract

Cadmium is an omnipotent environmental contaminant associated with the development of breast cancer. Studies suggest that cadmium functions as an endocrine disruptor, mimicking the actions of estrogen in breast cancer cells and activating the receptor to promote cell growth. Although acute cadmium exposure is known to promote estrogen receptor-mediated gene expression associated with growth, the consequence of chronic cadmium exposure is unclear. Since heavy metals are known to bioaccumulate, it is necessary to understand the effects of prolonged cadmium exposure. This study aims to investigate the effects of chronic cadmium exposure on breast cancer progression. A MCF7 breast cancer cell line chronically exposed to 10^−7^ M CdCl_2_ serves as our model system. Data suggest that prolonged cadmium exposures result in the development of more aggressive cancer phenotypes – increased cell growth, migration and invasion. The results from this study show for the first time that *chronic* cadmium exposure stimulates the expression of SDF-1 by altering the molecular interactions between ERα, c-jun and c-fos. This study provides a mechanistic link between chronic cadmium exposure and ERα and demonstrates that prolonged, low-level cadmium exposure contributes to breast cancer progression.

## Introduction

Breast cancer is one of the most common malignancies afflicting women in the United States, and it has the second highest mortality rate associated with any cancer. The majority of breast cancers initially develop as hormone-dependent, with estrogen receptors expressed in approximately 70% of breast cancer cases. The presence or absence of ERα is a key determinant of the prognosis of the disease, in addition to determining whether the cancer will respond to hormone therapy or not. ERα-positive breast cancers are often hormone-responsive and are typically treated with antiestrogens like tamoxifen. However, hormone-dependent breast cancer frequently progresses into more malignant cancer phenotypes that are often hormone-independent. In many cases, the estrogen receptor (ERα) is still present, but the role of ERα in hormone refractory breast tumorigenesis and its underlying mechanism are unclear. A potential mechanism involves metalloestrogens– heavy metals that function as endocrine disruptors and mimic the actions of estrogen.

Among the metalloestrogens, cadmium is the best characterized. Cadmium is derived from various industrial sources, including present and former mining activities, production of alloys, batteries, fertilizers, pigments, and combustion by-products. Human exposure results from the consumption of contaminated water and food or inhalation of cigarette smoke and contaminated fumes. Cadmium exposure has long been associated with the development of breast cancer [1]–[5], but the mechanism of cadmium’s action on breast cancer remains elusive. Recent studies have suggested that cadmium may function as an endocrine disruptor to perturb the normal hormonal cycle and promote neoplastic growth in the mammary gland [2], [4]. Animal studies have also shown that cadmium exposure can increase the density of the mammary glands as well as induce changes in the lining of the uterus, all of which are early signs of hormone-related tumorigenesis [4], [6], [7].

Cadmium has also been shown to promote ER-dependent breast cancer cell proliferation, potentially through the activation of the ER signaling pathway [3], [5], [8], [9]. Results from our lab and others have suggested that cells treated with cadmium express higher levels of ER target genes, including cyclin D1, c-myc, progesterone receptor, and cathepsin-D [3], [5], [8]. Although cadmium is known to bind to ERα, the mechanism behind ER-mediated gene expression and subsequent breast cancer cell growth is not completely established. Results from our previous study showed that cadmium promotes the nuclear localization of ERα and enhances the binding of ERα to target gene promoters [8]. It was also demonstrated that cadmium potentiates the interaction between ERα and c-jun, a member of the AP-1 family of transcription factors.

Although there is a better understanding of how acute cadmium exposure activates ERα and mediates the expression of genes associated with cell growth, it is unclear how *chronic* exposure of cadmium may affect breast cancer development and/or ER-target gene expression. Previous studies on prostate cancer and sarcomas have suggested that chronic exposure to cadmium is associated with the development of more malignant tumors [10]–[12]. Furthermore, cadmium has been detected in breast tumor tissues [1], [13]–[16]. While healthy individuals had measurable cadmium levels in their mammary glands, significantly higher levels of cadmium were found in patients with breast cancer [1], [13]–[16]. The effects of cadmium in these tumors and whether its presence results in further progression of the disease are unknown, underscoring the need for chronic exposure studies.

This study aims to investigate the chronic effects of cadmium exposure on breast cancer progression. We developed a series of cadmium exposed-breast cancer cell lines to study the effects of chronic cadmium exposure on breast cancer progression. This study provides several lines of evidence suggesting that prolonged cadmium exposure results in more aggressive cancer phenotypes– increased cell growth, migration and invasion. Our results also show for the first time that cells chronically exposed to cadmium express higher levels of SDF-1 in an ERα-dependent manner. Furthermore, data from this study also suggest that prolonged cadmium exposure alters the molecular dynamics between ERα and c-jun/c-fos to mediate direct changes in the expression of cancer-promoting genes like SDF-1.

## Materials and Methods

### Cell Culture

MCF7 cells were obtained from the American Type Culture Collection (ATCC, Manassas, VA). MCF7-Cd was developed by exposing parental MCF7 cells to 10^−7^ M CdCl_2_ for over 6 months. Parental MCF7 and MCF7-Cd were maintained in Dulbecco’s Modified Eagle Medium (DMEM) supplemented with 10% Fetal Bovine Serum (FBS; Hyclone, Logan, UT) and 1% penicillin and streptomycin (P/S). Both cell lines were subcultured every 3–4 days.

### Growth Assay

1.5×10^5^ MCF7 and MCF7-Cd (MCF7-Cd4, Cd7, and Cd12) cells were plated in six-well plates using hormone depleted media. Cells were counted in triplicate every 2 days using a cell counter (Nexcelom Bioscience LLC, Lawerence, MA). Data points represent three independent experiments.

### Derivation of Cadmium Cell Lines

To derive clonal cell lines chronically exposed to cadmium, MCF7 cells were first exposed to 10^−7^ M CdCl_2_ for over 6 months in order to mimic chronic exposures. Chronically exposed cells were plated on soft agar to allow single cells to develop into colonies. Briefly, a 1% agar solution made with complete media (DMEM+10% FBS +1% P/S) was used to create the bottom layer. Serial dilutions of single cell suspensions were mixed with enough complete media to make a 0.6% top agar that was added over the 1% bottom agar layer and allowed to solidify for 30 minutes at room temperature followed by incubation at 37°C. When colonies sizes reached about 100 cells or more, they were individually removed from soft agar under aseptic conditions and resuspended in complete media to permit further expansion.

### Scratch Wound Assay

Approximately 2.0×10^4^ cells were plated in 6-well plates and allowed to grow to approximately 75–80% of confluency in DMEM. A wound or scratch was created with a 200 µL micropipette tip and washed twice with 1X PBS. Cells were allowed to migrate for 3 and cells were visualized under 5X objective with a 10X ocular magnification (with a final magnification of 50X). At least three frames of each well were captured digitally at day 0 and day 3. Images presented in the figures are representative of three independent experiments done in triplicates.

### Migration and Invasion Assay

A modified Boyden chamber assay was used to quantitatively assess the cells’ migration and invasion abilities. For the migration assay, cells were hormone-deprived, and 5.0×10^4^ cells were seeded in the upper chamber over polycarbonate membrane inserts and allowed to migrate toward the lower chamber filled with DMEM+10% FBS+1% P/S. The cells were incubated for 12–18 hours, fixed in formalin, and stained with crystal violet. The number of cells that migrated to the underside of the filter was counted in triplicate. For the invasion assay, a CytoSelect™ Cell Invasion Assay Kit (Cell Biolabs, Inc, San Diego, CA) was used according to the manufacturer’s protocol. Briefly, 5.0×10^4^ hormone-deprived cells were seeded in the upper chamber and the cells that invaded through the matrigel were fixed, stained and counted as previously described.

### Western Blot

Cells were lysed in 1% SDS-HEPES buffer and the total protein concentration was normalized using the Bio-Rad Dc Protein Assay kit (Bio-Rad, Hercules, CA). Proteins were separated using SDS-polyacrylamide gel electrophoresis and transferred to polyvinylidene fluoride (PVDF) membranes (Millipore, Billerica, MA). Protein expression was monitored using protein-specific antibodies: α-ERα (NeoMarkers, Fremont, CA), α-cyclin D_1_ (Santa Cruz Biotechnology, Santa Cruz, CA), α-c-myc (Santa Cruz Biotechnology), α-cyclin A (Santa Cruz Biotechnology), α-cyclin E (Santa Cruz Biotechnology), α-cdk2 (Santa Cruz Biotechnology), α-cdk4 (BD Transduction Laboratories, San Jose, CA), α-p21 (Santa Cruz Biotechnology), α-p-27 (Santa Cruz Biotechnology), α-CDH1 (Santa Cruz Biotechnology), α-β-catenin (Cell Signaling Technology, Danvers, MA), α-GAPDH (Cell Signaling Technology).

### SDF-1 ELISA

Cells were plated at a density of 1×10^5^ cells per well in 6-well plates. Cell culture media was collected after 48 hours and stromal cell-derived factor-1 (SDF-1) levels were measured with enzyme-linked immunosorbent assays (ELISA) SDF-1 kit (RayBiotech, Inc, Norcross, GA) according to the manufacturer’s instructions. Briefly, 100 µL of sample was added to 96-well plates containing immobilized antibodies specific for human SDF-1 and allowed to incubate overnight with shaking at 4°C. Wells were then washed and a biotinylated anti-human SDF-1 antibody was added, followed by another wash and subsequently by the addition of HRP-conjugated streptavidin. Following a final wash, tetramethylbenzidine (TMB) substrate for color development was added and samples were read at 450 nm after the addition of a stop solution.

### siRNA Transfection

1×10^5^ cells were plated in 6 well plates and transfected with siRNA (Santa Cruz Biotechnology) targeting either ERα, c-jun or c-fos using siRNA transfection reagents (Santa Cruz Biotechnology). A scrambled siRNA was transfected as a control. Five hours after transfection, the medium was changed to DMEM medium containing 10% FBS and 1% P/S. Cells were harvested 48 hours later for gene and/or protein expression analysis using semi-quantitative reverse transcription-polymerase chain reactions (RT-PCR) or quantitative PCR (qPCR), and Western blot analysis, respectively.

### Reverse Transcriptase Polymerase Chain Reaction (RT- PCR)

Total RNA was isolated from cells using TRI-Reagent (Zymo Research Corporation, Irvine, CA) and columns from the Direct-zol RNA MiniPrep kit (Zymo Research) according to the manufacturer’s protocol. Three micrograms (µg) of total RNA were used for the reverse transcription reaction with oligo-dT_18_ primers and Moloney Murine Lukemia Reverse Transcriptase (M-MLV RT, Promega, Madison,WI). Gene expression was monitored using semi-quantitative PCR for 28 cycles. PCR products were separated by agarose gel electrophoresis, and visualized using Chemi-Doc (Biorad). Gene expression was quantified using gene specific primers with quantitative real-time PCR with SYBR green (SA Biosciences, Valencia, CA). All primers were synthesized by Integrated DNA Technologies, Inc. (IDT; San Diego, CA). RT-PCR Primer Sequences:

SDF-1_F_ : GTCAGCCTGAGCTACAGATGC SDF-1_R_: CACTTTAGCTTCGGGTCAATG

ERα_F_: ATGACCCTCCACACCAAAGCAT ERα_R_: ACTGGCCAATCTTTCTCTGCCA

c-myc_F_: CTCCACACATCAGCACAACT c-myc_R_: GTTTCCGCAACAAGTCCTCT

cycD1_F_: AATGTGTGCAGAAGGAGGTC cycD1_R_: GAGGGCGGATTGGAAATGAA

GAPDH_F_: GAAATCCCATCACCATCTTCCAG GAPDH_R_: ATGAGTCCTTCCACGATACCAAAG

### Coimmunoprecipitation (CoIP) Assay

Cells were plated in 10 cm tissue culture plates and harvested 48 hours later. Cells were lysed in 0.5% NP-40 lysis buffer and sonicated three times for 30 seconds. Cell lysates were immunoprecipitated with α-ERα, α-c-fos, α-c-jun or normal rabbit IgG (Santa Cruz Biotechnology) for 2 hours, followed by incubation with either protein A or G agarose beads for 1 hour. Protein complexes were separated with SDS-PAGE followed by Western blot analysis with ERα, c-jun, c-fos, and normal rabbit IgG antibodies.

### Chromatin Immunoprecipitation (ChIP) Assays

Cells were plated in 10 cm plates and harvested by fixation with formaldehyde. Cells were lysed and sonicated in lysis buffer to fragment the chromatin into 1–2 kb fragments. Chromatin mixtures were immunoprecipitated with antibodies specific for ERα, c-fos, and c-jun. Antibody complexes were purified using protein-A agarose beads (Pierce, Rockford, IL). After DNA-protein complexes were reversed cross-linked (65°C for 4–6 hours), DNA fragments were purified using QIAQuick columns (Qiagen, Germantown, MD). Occupancy at specific promoters were determined with PCR using promoter specific primers. For the re-ChIP assay, the protein A-antibody complexes from the first ChIP assay were extracted with 200 µl of 10 mM DTT for 30 minutes at 37°C. After the supernatant was diluted 10X with re-ChIP buffer (20 mM Tris- HCl, pH 8.1, 2 mM EDTA, 150 mM NaCl, and 0.1% Triton X-100), the chromatin mixture was re-ChIPed with a second antibody: α-ER, α-c-jun, α-c-fos or normal rabbit IgG, (Santa Cruz Biotechnology). This step was repeated one more time for the triple-ChIP assay and the re-ChIPed DNA-protein complexes were immunoprecipitated with a third antibody (c-fos or rabbit IgG). Following, the purification of the final antibody complexes with protein A beads, the DNA was reverse-cross-linked from the protein and purified as described above.

ChIP Primer Sequences:

SDF1-ChIP_F_: CACCATTGAGAGGTCGGAAG [17]

SDF1-ChIP_R_: AATGAGACCCGTCTTTGCAG [17]

cyclin D1-ChIP_F_: CATTCAGAGGTGTGTTTCTCCC

cyclin D1-ChIP_R_: CTCAGCGACTGCATCTTCTTTC

c-myc-ChIP _F_:GACACATCTCAGGGCTAAACAG

c-myc-ChIP _R_: GAGAGTGGAGGAAAGAAGGGTA

U6RNA-ChIP_F_: GAGGGCCTATTTCCCATGATTC

U6RNA-ChIP_R_ :GAATTTGCGTGTCATCCTTGC

### Luciferase Reporter Assay

The reporter gene assay was performed by sequentially transfecting MCF7 cells with siRNA targeting ERa (Santa Cruz Biotechnology) followed by the SDF1 promoter-Luc reporter plasmid. Five hours after siRNA transfection, the second transfection with the SDF-1 Luc reporter plasmid along with the pRL-SV40 *Renilla* luciferase plasmid (Promega) was accomplished using Fugene HD (Promega) according to the manufacturer’s protocol. The medium was changed to DMEM media containing 10% FBS and penicillin and streptomycin, and cells were harvested 48 hours post-transfection and analyzed using a dual luciferase assay kit (Promega). All reporter gene assays were performed in quadruplicate, with the entire experiment repeated at least three times. SDF-1-Luc was purchased from Genecopoeia (Rockville, MD).

## Results

**Figure 1:**
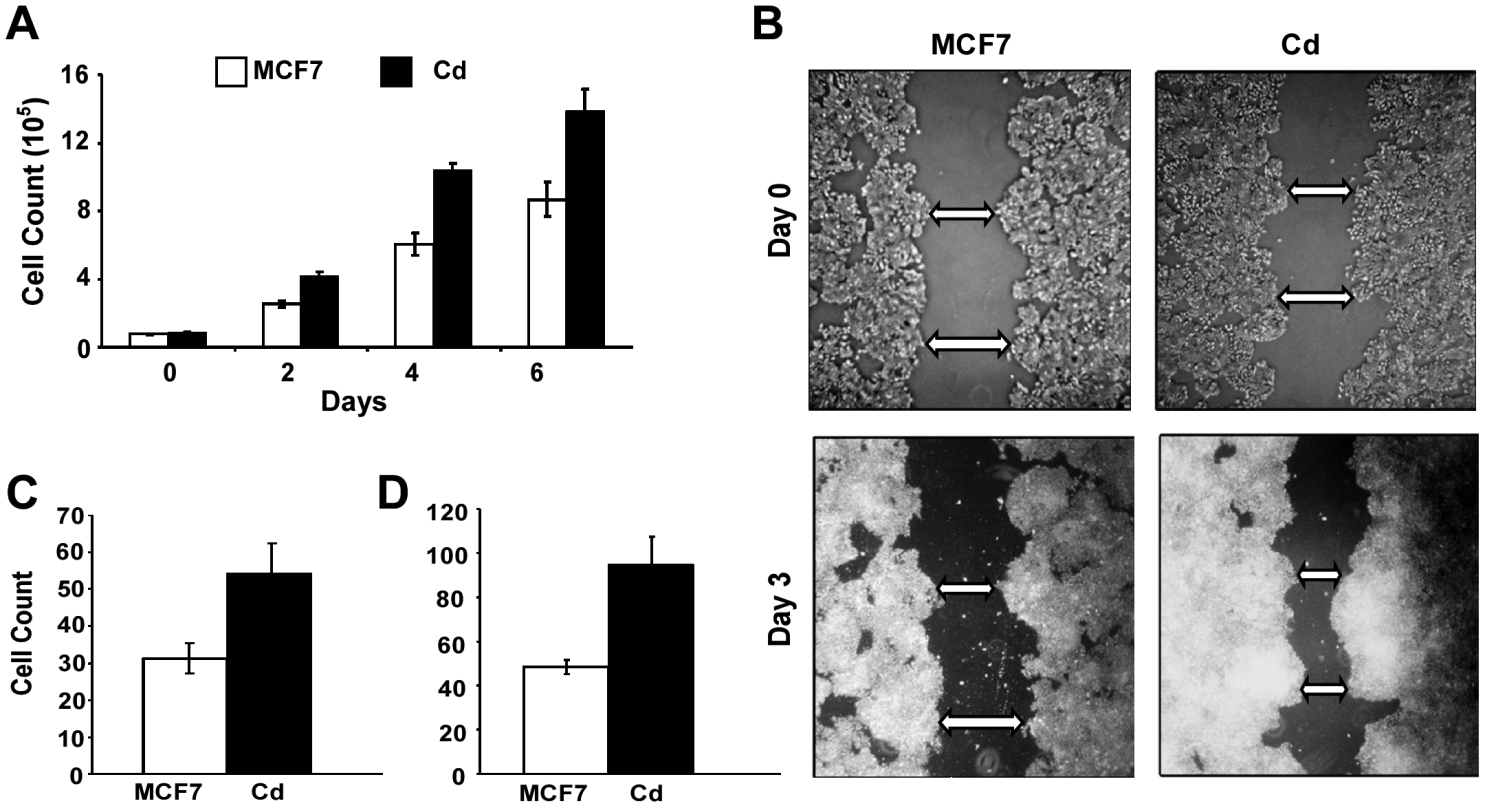
Chronic cadmium exposure induces more aggressive cancer phenotypes: cell growth, migration and invasion. (**A**) MCF7-Cd cells (black bars) and parental MCF7 cells (white bars) were plated in 6 well plates under hormone-deprived conditions and cell growth was monitored 2, 4, and 6 days after plating by counting in triplicate. Experimental results are averages of three independent experiments (P<0.01). (**B**) A scratch wound assay was used to assess cell migration ability. MCF7 and MCF7-Cd cells were plated in 6-well plates and allowed to reach 70–80% confluence. A scratch was created using a p-200 pipette tip and a digital image was captured on Day 0. Cells were then allowed to grow and migrate for 3 days and images of the wound were captured 3 days later. Images are representative of three independent experiments done in triplicates. Arrows are indicating the same region on the scratch on day 0 and day 3. (**C**) Modified Boyden chamber assays were performed to measure the migration abilities of the cells. MCF7 (white bars) and MCF7-Cd (black bars) cells were seeded into upper chambers and cells were allowed to migrate to lower chamber for 16 hours. Data is representative of 3 independent experiments performed in triplicate (P<0.01). (**D**) An invasion assay was performed by seeding either MCF7 (white bars) or MCF7-Cd (black bars) cells in the upper chambers. Cells were allowed to invade through matrigel-coated membranes for 18 hours. Data is an average of 3 independent experiments done in triplicate (P<0.01).

We previously found that acute cadmium exposures increase growth rates of three ERα-positive breast cancer cell lines– MCF7, T47D and ZR75-1– and increase the expression of genes associated with growth [8]. The observation that acute cadmium exposure stimulates breast cancer cell proliferation led us to question the effects of chronic cadmium exposure on breast cancer progression. To understand how chronic cadmium exposure affects the progression of ER-positive breast cancer, a cell line chronically exposed to cadmium was developed by exposing MCF7 cells to low concentrations of cadmium (10^−7^ M) for over six months. The growth properties of cells chronically exposed to cadmium (MCF7-Cd) were analyzed in the presence or absence of cadmium and compared to parental MCF7 cells. Approximately 1.5×10^4^ cells were plated in 6-well plates under hormone-deprived conditions and treated with 10^−7^ M cadmium chloride (CdCl_2_) or mock treated with phosphate buffered saline (PBS). Cell growth was monitored 2, 4 and 6 days after treatment. The MCF7-Cd cells displayed a significantly faster growth rate than the parental MCF7 cells in the presence and absence of cadmium (Fig. 1A).

Metastatic phenotypes including the ability to migrate and invade through extracellular matrix were also evaluated. The ability of cells chronically exposed to cadmium to migrate was analyzed and compared to parental MCF7 cells using a scratch-wound assay (Fig. 1B). Cells were plated in a 6-well plate and allowed to reach 70–80% confluence; a scratch was created using a p200 pipette tip. Cells were rinsed with PBS and migration was observed 3 days later. Results in figure 1B suggest that MCF7-Cd cells display a greater ability to migrate in comparison to parental MCF7 cells. To confirm the qualitative observations in figure 1B, a modified Boyden Chamber assay was used to quantify the difference in cell migration between MCF7 and MCF7-Cd cells (Fig. 1C). In this assay, 5×10^4^ cells (MCF7 or MCF7-Cd) were plated in the upper chamber and allowed to migrate for 16 hours through an 8 µM polycarbonate membrane. Cells that have the ability to migrate and attach to the underside of the membrane were fixed and stained with crystal violet, and the number of migrated cells were visualized and counted using a microscope. Results in figure 1C further demonstrate that cells chronically exposed to cadmium display an increased ability to migrate (31 vs. 54, p<0.01). The invasive ability of the MCF7-Cd cells was also investigated using a similar assay, except the membranes in the chambers were coated with matrigel in order to mimic the extracellular matrix (Fig. 1D). Again, 5×10^4^ cells (MCF7 or MCF7-Cd) were added to the upper chamber, and cells with the ability to invade had to digest the matrigel and attach to the underside of the chamber. Consistent with the results from the migration assay, MCF7-Cd cells displayed a greater ability to digest and invade through the matrigel (48 vs 94, p<0.01).

**Figure 2:**
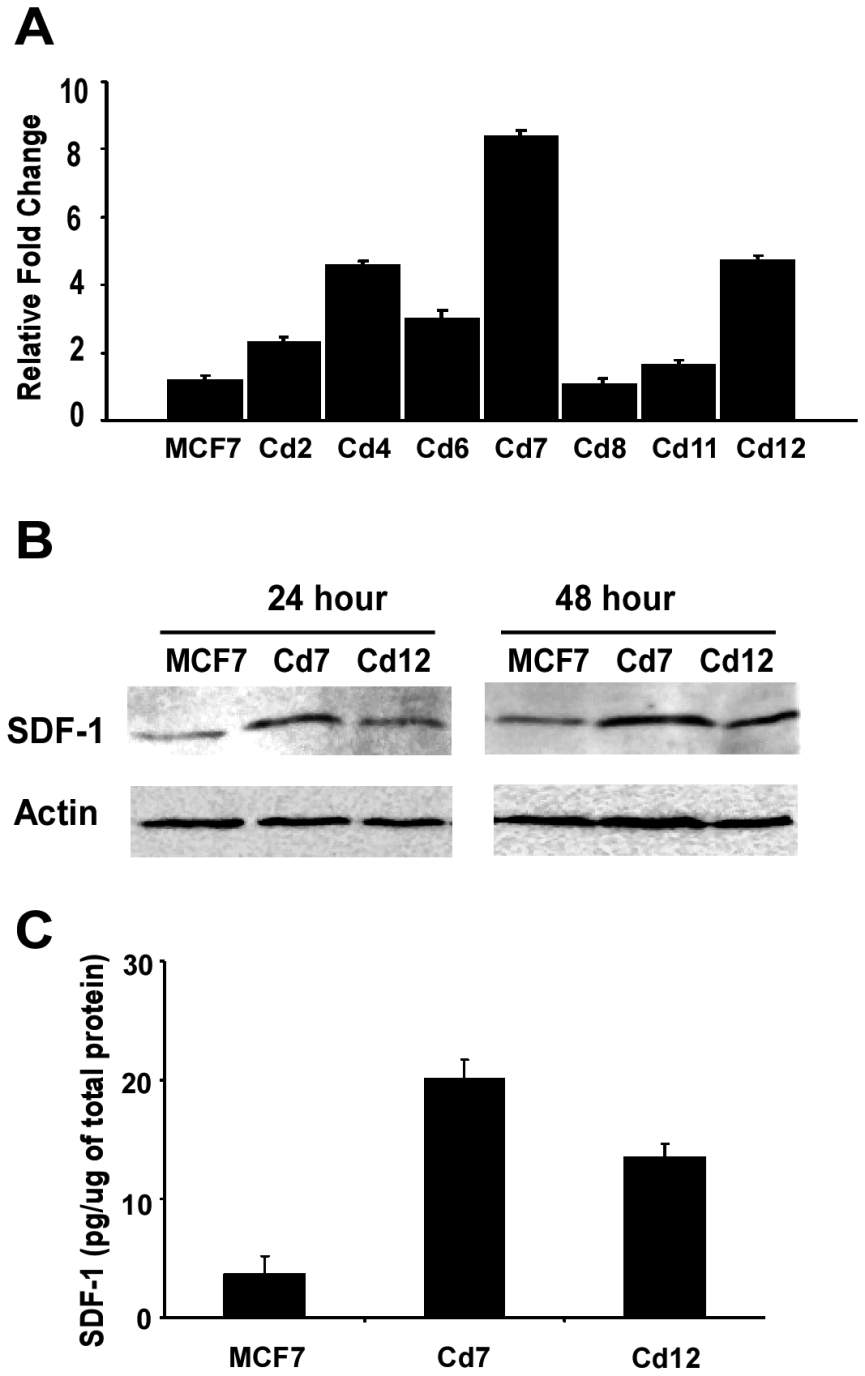
Cells chronically exposed to cadmium express and secrete higher levels of SDF-1. (**A**) MCF7 and MCF7-Cd clonal derivatives (Cd-2, -4, -6, -7, -8, -11 and -12) were plated in 6 well plates and total RNA was isolated for gene expression analysis using quantitative PCR. Data is presented as relative SDF-1 fold increases with MCF7 as the control. (**B**) SDF-1 protein expression in MCF7, Cd7, and Cd12 cells was analyzed with Western blot analysis. Actin was used as a loading control. (**C**) Conditioned media was collected in the same experiments shown in B after 24 hours, and the levels of SDF-1 were measured with an enzyme-linked immunosorbent assay (ELISA). Data is representative of several experiments done in triplicate (P<0.01).

To understand the molecular alterations that occur in the cells exposed for prolonged periods to cadmium, clonal cell lines (MCF7-Cd2 to Cd12) were derived from the pool of MCF7-Cd cells. After confirming that the cadmium-adapted cell lines displayed the same characteristics as observed in figure 1– increased growth, migration and invasion (Fig. S1), we analyzed the differential gene expression of these cells using both a metastasis-specific PCR array (Superarray RT-Profiler) and microarray. Both array studies identified SDF-1, also known as CXCL12, as being up-regulated in a majority of the cadmium-adapted cells. Further verification using quantitative RT-PCR demonstrated that about 70% of the cadmium-adapted cell lines expressed higher levels of SDF-1 (Fig. 2A), with clones Cd7 and Cd12 expressing the highest levels. The levels of SDF-1 protein present in the Cd7 and Cd12 cells and secreted into the media were also examined using Western blot analysis and ELISA, respectively. Consistent with the gene expression data, levels of SDF-1 in both the protein lysates and in the media were elevated, as shown in figures 2B and C.

**Figure 3:**
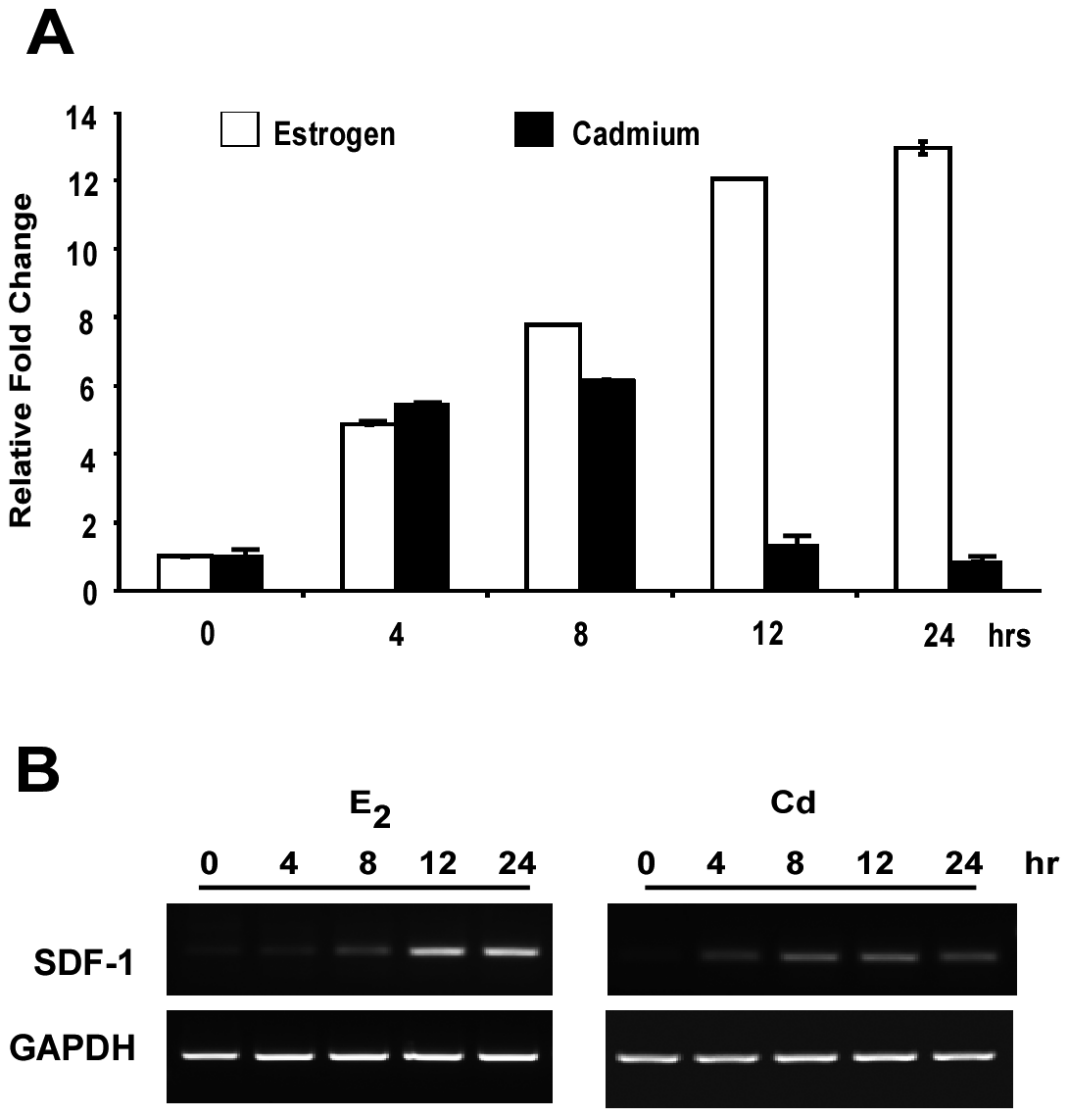
SDF-1 is induced by acute cadmium and estrogen exposure. MCF7 cells were hormone deprived and treated with either 10^−7^ M E_2_ (white bars) or 10^−6^ M CdCl_2_ (black bars). Cells were harvested for gene expression analysis using (**A**) quantitative and (**B**) semi-quantitative RT-PCR. Quantitative PCR data is presented as relative SDF-1 fold increases.

Interestingly, SDF-1 has been shown to be an estrogen receptor (ERα) regulated gene [18]. Since cadmium functions as a metalloestrogen, we questioned whether cadmium could induce the expression of SDF-1. To determine if the expression of SDF-1 is regulated by acute cadmium exposure, parental MCF7 cells were treated with either 10^−6^ M CdCl_2_ or 10^−7^ M 17β-estradiol. Results in figure 3 indicate that both cadmium and estrogen can increase SDF-1 expression by 6 and 12 fold, respectively (Fig. 3A–B). Estrogen-induced SDF-1 expression continued to increase for up to 24 hours, whereas cadmium-induced SDF-1 expression peaked after 8 hours of exposure. Although, cadmium induction was not as robust as 17β-estradiol induction, these results clearly demonstrate that cadmium has the ability to stimulate SDF-1 expression within hours of exposure.

**Figure 4:**
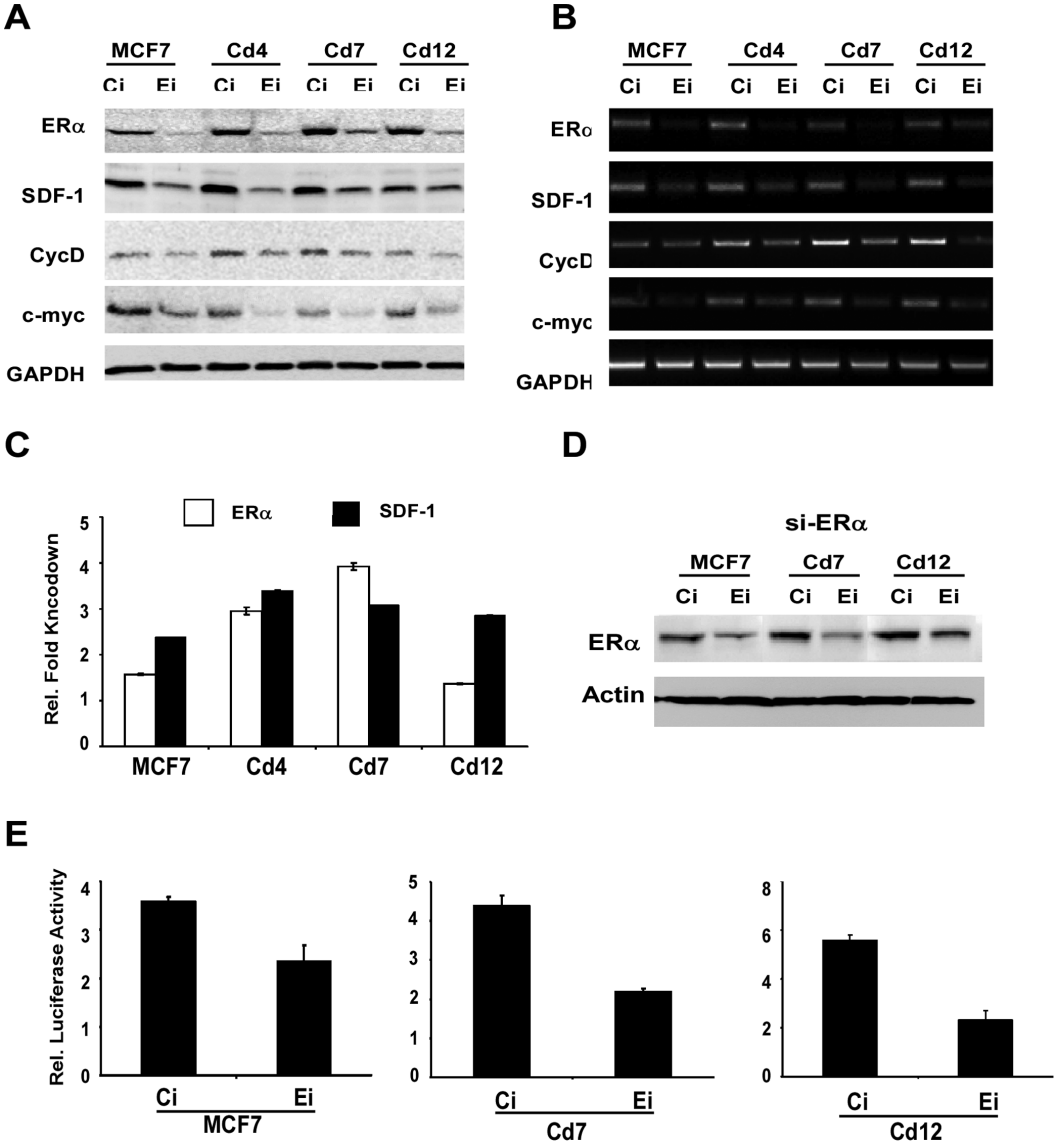
SDF-1 expression in cells chronically exposed to cadmium is dependent on ERα. MCF7, Cd4, Cd7 and Cd12 cells were plated in 6-well plates and transfected with either si-ERα (Ei) or si-control (Ci), and collected 48 hours after treatment for protein and gene expression analyses using (**A**) Western blot and (**B**) RT-PCR, respectively. Changes in gene expression were also measured using (**C**) quantitative RT-PCR, and fold knockdown was expressed as ΔCt^Ei^/ΔCt^Ci^. Fold knockdown of ERα and SDF-1 are represented in white and black, respectively. Data is representative of at least three independent experiments done in triplicate. GAPDH was used as a control. MCF7, Cd7 and Cd12 were also transfected with siRNA targeting ERα and the transactivation of the SDF-1 promoter measured using a luciferase reporter assay. Following transfection, cells were collected for (**D**) Western blot analysis and (**E**) reporter gene assay. Data represents at least three independent experiments done in quadruplicate. Actin was used as a loading control for the Western blot.

Given that SDF-1 is an ER-target gene, SDF-1 expression was further analyzed in the Cd-cell lines to see if it was dependent on ERα by depleting endogenous levels of ERα (Fig. 4). To mediate ERα silencing (Ei), siRNA was transfected into parental MCF7, Cd4, Cd7 and Cd12 cells, and the effects of decreased ERα levels on SDF-1 expression were examined. Cells transfected with scrambled siRNA were used as controls (Ci). At 48 hours after transfection, cells were collected for both protein and gene expression analyses using Western blot and semi-quantitative RT-PCR, respectively (Fig. 4A–B). Gene expression analysis was also confirmed by quantitative RT-PCR (qRT-PCR) (Fig. 4C). As expected, the successful depletion of ERα by siRNA resulted in lower levels of SDF-1 in parental MCF7 cells. Interestingly, a similar effect was observed in cells chronically exposed to cadmium, suggesting that SDF-1 expression in the cadmium-adapted cells is also dependent on ERα. To determine if ERα plays a direct role in regulating the transcription of SDF-1, a luciferase reporter assay was used in the presence and absence of ERα (Fig. 4E). MCF7, Cd7 and Cd12 cells were transfected with either a control siRNA (Ci) or one that targets ERα (Ei). The depletion of ERα, as shown by the Western blot (Fig. 4D), significantly decreases the transactivation of the SDF-1 promoter in all three cell lines (Fig. 4E), further establishing the role of ERα in regulating the expression of SDF-1.

**Figure 5:**
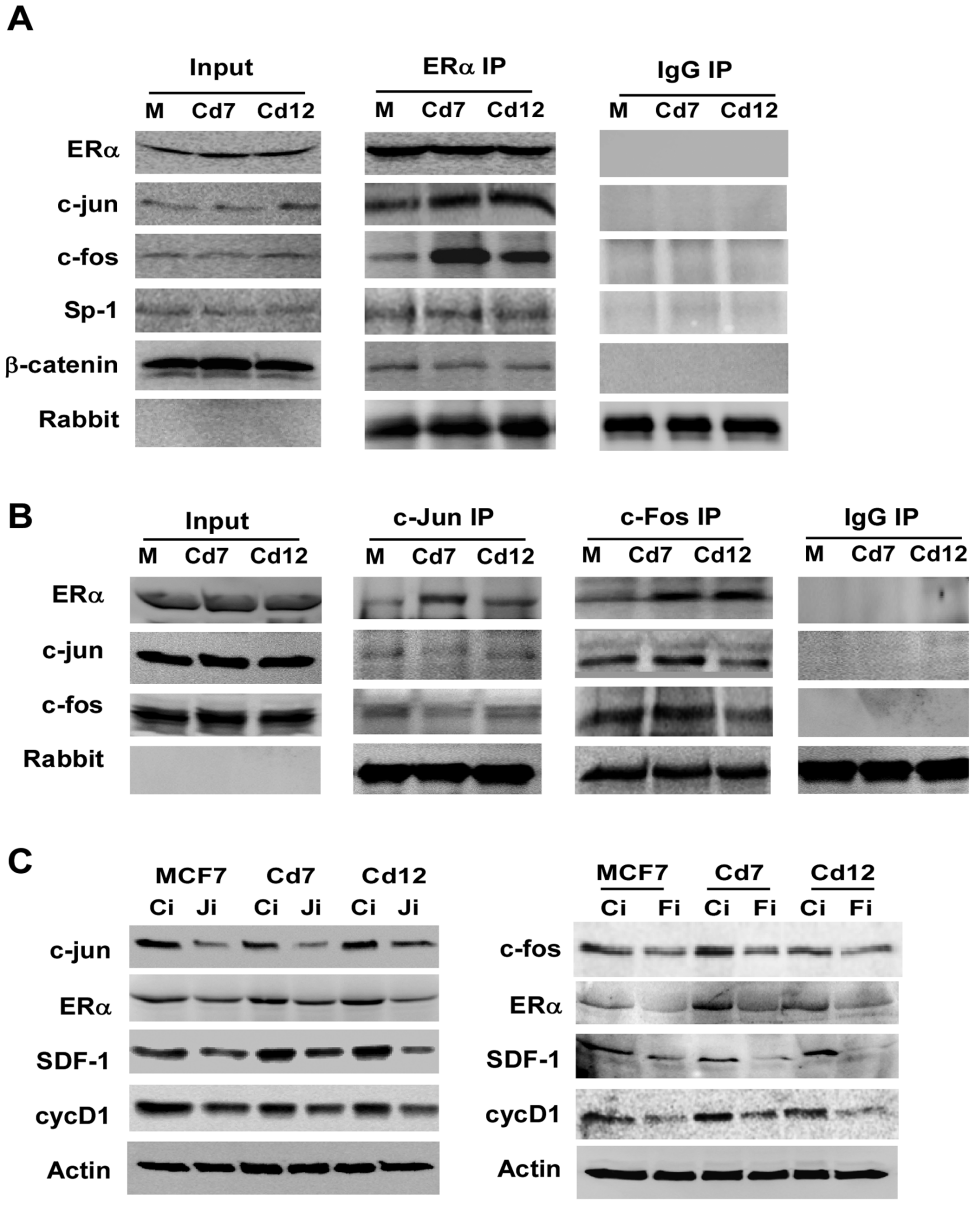
Prolonged exposure to cadmium enhances the interactions of ERα with c-jun and c-fos. (**A**) MCF7, Cd7 and Cd12 cell lysates were immunoprecipitated with either α-ERα or normal rabbit IgG. Proteins interacting with ERα were analyzed with Western blot analysis. (**B**) Reverse co-IP was performed with α-c-jun, α-c-fos, or normal rabbit IgG. (**C**) MCF7, Cd7 and Cd12 were plated in 6-well plates and transfected with siRNA targeting either c-jun (Ji), c-fos (Fi) or a scramble siRNA control (Ci) and collected 48 hours later for protein analysis.

Although SDF-1 is an ERα-regulated gene, it is unclear whether ERα regulates SDF-1 alone or in collaboration with other transcription factors. To determine if ERα interacts with other transcription factors, a co-immunoprecipitation experiment was used to identify potential transcription factors that may interact with ERα in cells chronically exposed to cadmium (Fig. 5). Cells were grown for 48 hours, and cell lysates were precipitated with α-ERα antibodies. The presence of specific transcription factors in the immunoprecipitated complexes was determined with Western blot analysis. Results in figure 5A indicate that a greater fraction of c-fos and c-jun co-precipitated with ERα in the Cd7 and Cd12 cells when compared to the parental MCF7 (M) cells, whereas similar levels of Sp-1 and β-catenin– two transcription factors known to interact with ERα– were found in both parental MCF7 and cadmium-adapted cells (Fig. 5A). Changes in the interaction of ERα with c-jun and c-fos were further confirmed by reciprocal co-immunoprecipitation with antibodies against c-jun and c-fos (Fig. 5B).

Despite the observation that ERα co-precipitates with both c-jun and c-fos, it is unclear whether these AP-1 transcription factors are directly involved in the regulation of SDF-1. To understand the role of c-jun and c-fos in mediating the expression of SDF-1, the expression of SDF-1 cells was analyzed following siRNA-mediated silencing of c-jun and c-fos (Fig. 5C). MCF7, Cd7 and Cd12 cells were transfected with siRNA to target either c-jun (Ji) or c-fos (Fi), and cells were harvested 48 hours later for Western blot analysis (Fig. 5C). While lower levels of c-jun and c-fos resulted in a significant reduction of SDF-1 expression, these lower levels of c-jun and c-fos apparently led to decreased levels of ERα, complicating the determination of whether c-jun and c-fos directly regulate SDF-1 expression.

**Figure 6:**
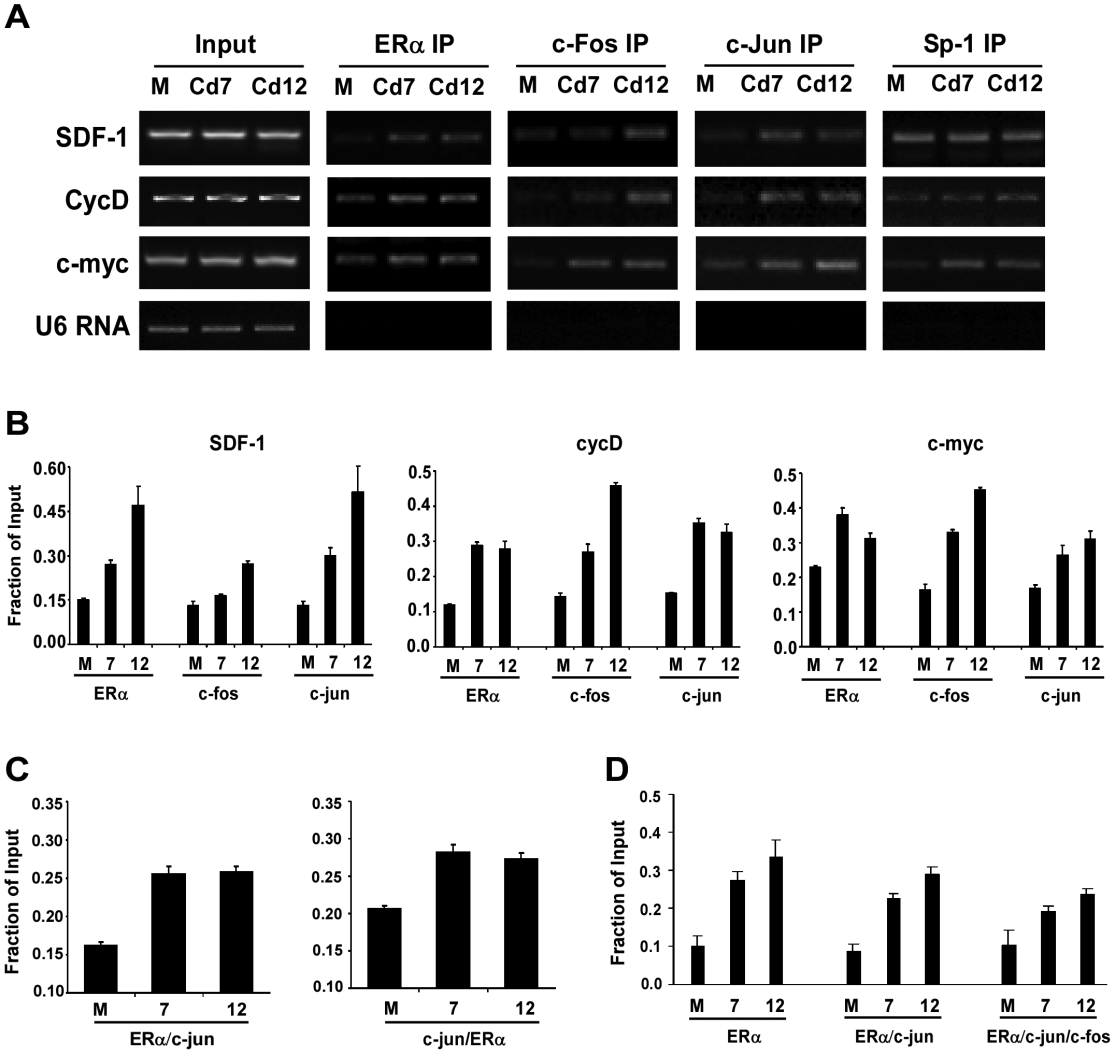
ERα, c-fos and c-jun are recruited to SDF-1 promoter. (**A**) MCF-7, Cd7 and Cd12 cells were harvested for chromatin immunoprecititation (ChIP) analysis. ChIP analysis was done with α-ERα, α-c-fos, α-c-jun, and α-Sp1, and recruitment of proteins to the SDF-1, cyclin D1 and c-myc promoters was determined using promoter specific primers and semi-quantitative PCR. (**B**) Band intensities of PCR products for SDF-1, cycD, and c-myc were quantified and normalized to input using Quantity One (Bio-Rad) (P<0.001). (**C**) In the ChIP re-ChIP assay, DNA/protein complexes from the first ChIP assay were extracted and re-ChIPed with a second antibody. The occupancy of ERα/c-jun or c-Jun/ERα complexes on the SDF-1 promoter was analyzed as in B (P<0.001). (**D**) To confirm the presence of c-fos in the ERα/c-jun complex, reChIPed DNA containing ERα/c-jun complexes were immunoprecipitated with a third antibody, α-c-fos, and the resulting DNA fragments were analyzed as in B (P<0.001).

Since the regulatory region of SDF-1 consists of only one full ERE site located 234 bp upstream of the promoter, five half ERE sites located within the proximal promoter, and multiple AP-1 and Sp-1 sites on the promoter [17], it is probable that ERα may interact with other transcription factors to regulate SDF-1 expression. Therefore, a chromatin immunoprecipitation (ChIP) assay was used to determine whether a greater fraction of c-jun and c-fos is recruited to the SDF-1 promoter in cells chronically exposed to cadmium. Cells were plated for 48 hours and harvested for ChIP analysis. The recruitment of ERα, c-jun and c-fos was analyzed by PCR. Results in figures 6A and B indicate that cells chronically exposed to cadmium (Cd7 and Cd12) had higher levels of ERα recruited to the SDF-1 promoter in comparison to parental MCF7 (M) cells (27% and 47% vs. 15%, respectively). Similarly, the occupancy of c-jun and c-fos on the SDF-1 promoter were significantly elevated in Cd7 and Cd12 cells when compared to parental MCF7 cells, with c-jun showing at least a 2-fold increase (Fig. 6A–B). The occupancy of all three transcription factors (ERα, c-jun, and c-fos) were also found elevated in the cyclin D1 and c-myc promoters of cells chronically exposed to cadmium (Fig. 6A–B). In contrast, Sp-1 was only found elevated on the c-myc promoter of cells chronically exposed to cadmium (Cd7 and 12); no significant difference in Sp-1 levels was observed on the SDF-1 and cyclin D1 promoters among the different cell lines (Fig. 6A).

While the results from the ChIP assay strongly support the notion that ERα, c-jun and c-fos all regulate SDF-1 expression, it is unclear whether these transcription factors work independently or together as one regulatory complex. To fully understand the components of the transcription complex regulating SDF-1 expression, a ChIP re-ChIP assay was performed. Following the first ChIP assay with either ERα or rabbit IgG as a control, the DNA-protein complexes were extracted and re-ChIPed with a second antibody (c-jun, or rabbit IgG). Data from both forward and reverse ChIP re-ChIP assays display higher levels of ERα/c-jun complex bound to the SDF-1 promoter in Cd7 and Cd12 cells when compared to parental MCF-7 cells (Fig. 6C). Since previous studies have suggested that ERα and c-fos do not interact directly [19], we evaluated the presence of c-fos in the ERα/c-jun complex using a triple-ChIP assay (three sequential chromatin IPs), in which the re-ChIPed DNA containing ERα/c-jun complexes was immunoprecipitated with a third antibody (c-fos or rabbit IgG) to verify the presence of c-fos in the complex. Results in figure 6D not only confirm the occupancy of ERα/c-jun complex on the SDF-1 promoter, but also demonstrate that c-fos is involved in transcriptional regulation of SDF-1 in both parental and cadmium-adapted cell lines.

## Discussion

Often referred to as an endocrine disruptor, cadmium is an environmental contaminant that has been shown to have the ability to bind to the estrogen receptor and alter the expression of various estrogen receptor target genes [3], [5], [8], [9]. While epidemiological and animal studies [3], [4], [6], [7], [20]–[23] have implicated cadmium as a carcinogen, there is no direct evidence suggesting cadmium promotes breast cancer in humans. Despite this, the International *Agency for Research on Cancer (*IARC) has classified cadmium as a potential human carcinogen. Although multiple studies– including work from this lab– have demonstrated that acute cadmium exposures can stimulate breast cancer cell growth and can activate the estrogen receptor to mediate the expression of genes associated with cell growth [2], [3], [5], [24]–[26], less is known about how chronic exposures of cadmium may contribute to the development and progression of breast cancer. Studies on prostate cancer and sarcomas have indicated that chronic exposures to cadmium can be associated with more malignant tumors [10]–[12]. Additionally, significantly higher levels of cadmium have been found in breast tumor tissues in comparison to non-tumor tissues [1], [13]–[16]. The chronic effects of cadmium on the progression of breast cancer and the molecular alterations induced by cadmium were never before fully elucidated.

Results from this study confirm that chronic cadmium exposure promotes the development of more aggressive cancer characteristics including increased cell growth and an increased ability to migrate and invade (Fig. 1). To understand the impact of chronic cadmium exposures at the molecular level, clonal cell lines were developed from the original MCF7-Cd cell line (MCF7-Cd2 to 12), and it was found that SDF-1, one of the genes identified in microarray expression analysis, was elevated in more than 70% of the cadmium-adapted cell lines (Fig. 2A). In addition to expressing higher levels of SDF-1 at the mRNA level, cells chronically exposed to cadmium (MCF7-Cd7 and Cd12) also expressed and secreted more SDF-1 protein into the media (Fig. 2B–C), consistent with its function as a chemokine, a factor that promotes cell migration and metastasis [27].

Interestingly, past studies have also demonstrated SDF-1 to be regulated by ERα [17], [18], [28]. Consistent with these findings, SDF-1 mRNA levels were indeed shown to increase within hours of the cells being treated with 17β-estradiol (Fig. 3A–B). Strikingly, our results demonstrate that the metalloestrogen, cadmium can also stimulate SDF-1 gene expression, but to a lesser extent than 17β-estradiol (6 vs.12 fold, Fig. 3). To establish a link between chronic cadmium-induced SDF-1 expression and ERα, siRNA was used to reduce intracellular levels of ERα in parental MCF7, MCF7-Cd4, -Cd7, and -Cd12 cells. Results showed that depleting ERα expression subsequently inhibited the expression of SDF-1 in both MCF7 and cadmium-adapted cells, suggesting that elevated SDF-1 expression is in part dependent on ERα (Fig. 4). In order to determine whether ERα regulates the transcription of the SDF-1 gene, a luciferase reporter assay was conducted using SDF1-*luc* and co-transfected siRNAs to silence ERα. Results in figure 4E suggest that depletion of ERα, as indicated by Western blot analysis (Fig. 4D), significantly reduces transcriptional activation of the SDF-1 promoter. Similarly, silencing ERα in cadmium-adapted and parental MCF-7 cells also decreased other ERα target genes, specifically cyclin D1 and c-myc (Fig. 4). This further indicates that the estrogen receptor signaling pathway is intact and may have a heightened response in cells chronically exposed to cadmium.

To determine if chronic cadmium exposure enhances the interaction of ERα with other transcription factors, co-immunoprecipitation experiments were performed. Results in figure 5A show that a greater fraction of c-jun and c-fos co-precipitated with ERα in the cadmium-exposed cells. These interactions were confirmed by reciprocal co-immunoprecipitation (co-IP) with either c-jun or c-fos (Fig. 5B). While the interaction of ERα with c-jun is well-documented [8], [19], [29], the direct interaction between ERα and c-fos has not been documented. A study by Teyssier et al. demonstrated that ERα binds directly to c-jun, but not to c-fos, in pull-down assays [19]. Although our co-IP results show an ERα/c-fos interaction, it is important to note that this interaction may be mediated indirectly by c-jun, especially since c-jun and c-fos often function as heterodimers [30]. Consistent with the results presented in this study, previous studies have demonstrated that both cadmium and arsenic can increase the interactions of ERα with c-jun under acute exposures [8], [29].

Though promising, the increased interactions between ERα, c-jun and c-fos do not prove that these complexes are directly involved in regulating the expression of SDF-1 in cells chronically exposed to cadmium. We attempted to show this via siRNA-mediated silencing of c-jun and c-fos, which did result in a reduction of SDF-1. Unfortunately, the data could not clearly demonstrate whether the decrease in SDF-1 was due solely to c-jun or c-fos since the down regulation of c-jun and c-fos also decreased endogenous ERα levels, which is already known to affect SDF-1 levels (Fig. 5C).

To confirm the roles of ERα, c-jun and c-fos in the transcriptional regulation of SDF-1 in cells chronically exposed to cadmium, chromatin immunoprecipiation (ChIP) assays were then used to determine the occupancy status of each transcription factor on the SDF-1 promoter. In line with previous reports by Zhu et al. and Boudot et al., our results demonstrate that ERα is recruited to the SDF-1 promoter [17], [28], and the promoter occupancy by ERα is elevated in cells chronically exposed to cadmium (Fig. 6A–B). Additionally, a greater fraction of c-jun and c-fos were also found on the SDF-1, cyclin D1, and c-myc promoters after chronic cadmium exposure. In contrast, the levels of transcription factor Sp-1 were only elevated on the c-myc promoter, suggesting that the cadmium-induced effects are promoter specific.

Whether ERα regulates the expression of SDF-1 by binding to classical ERE sites or via interactions with other transcription factors was uncertain. However, based on the occupancy patterns of c-jun and c-fos on the SDF-1 promoter, it seemed conceivable that SDF-1 is regulated in a similar manner as cyclin D1 and c-myc– both of which are regulated by the interaction of ERα with c-jun/c-fos heterodimers [31]–[35]. Since the interaction between ERα and c-fos is likely mediated indirectly by c-jun [30], further studies were carried out using both ChIP re-ChIP and triple-ChIP assays to determine if ERα regulates SDF-1 via interactions with c-jun/c-fos heterodimer. Data from both forward and reverse ChIP re-ChIP assays suggested that cells chronically exposed to cadmium (Cd7 and Cd12) exhibited higher levels of ERα/c-jun complexes on the SDF-1 promoter (Fig. 6C). The increase in ERα/c-jun interactions further emphasizes the importance of ERα and c-jun in regulating SDF-1 expression, especially in cells chronically exposed to cadmium. Consistent with these results, c-jun has been shown to regulate SDF-1 expression in other cell types [36], [37]. These new results also mirrored those from our previous study, which had demonstrated that the ERα/c-jun interaction was important in modulating the expression of genes in response to *acute* cadmium exposure [8]. Interestingly, the ERα/c-jun interaction has also been shown to be important in cells exposed to arsenic stress [29], suggesting this may serve as a common metal-response mechanism.

In summary, this study is the first to demonstrate that chronic cadmium exposure promotes the acquisition of more aggressive cancer phenotypes (e.g. growth, migration and invasion) by stimulating the expression of SDF-1, and altering the molecular interactions between ERα, and c-jun/c-fos. In essence, our data suggest that chronic cadmium exposure significantly alters the molecular dynamics of cells. We speculate that the molecular interactions of ERα with other transcription factors– including other members of the AP-1 family, E2F, and NFκB families– are also affected by chronic cadmium exposure, most likely in a promoter-specific manner [38], [39]. Since estrogen receptor is known to interact with many coactivators and corepressors [35], [39], it would also be interesting in the future to examine how chronic cadmium exposure may alter the molecular interactions between ERα and its coregulators. Additionally, we believe that studying the effects of chronic cadmium exposure on other cell types– both ERα positive and negative– will be crucial for further understanding cadmium-induced carcinogenesis. The results presented in this study offer molecular insights into how mammary tumors that have gradually bioaccumulated significant levels of cadmium can become more aggressive, and thus underscore the importance of studying the molecular effects of *chronic* cadmium exposure in breast carcinogenesis.

## Supporting Information

Figure S1 **Cadmium-adapted cells display more aggressive cancer phenotypes: cell growth, migration and invasion. (A)** MCF7-Cd clones (Cd4, Cd7, and Cd12) and parental MCF7 cells were plated in 6 well plates and growth was monitored 2, 4, and 6 days after plating by counting in triplicates with an automatic cell counter (P<0.01). Experimental results are representative of three independent experiments. **(B)** Modified-Boyden chamber assay was performed to measure the migration abilities of MCF-Cd cells. MCF7 (white bars), Cd7 (gray bars) or Cd12 (black bars) cells were seeded in upper chamber containing hormone-deprived media and allowed to migrate for16 hours to lower chamber containing media contain fetal bovine serum (FBS). Data is representative of 3 independent experiments done in triplicates (P<0.01). **(C)** An invasion assay was performed by seeding either MCF7 (white bars), Cd7 (gray bars) or Cd12 (black bars) cells in the upper chamber. Cells were allowed to invade through matrigel-coated membrane for 18 hours. Data is representative of 3 independent experiments done in triplicates (P<0.01).
(TIF)
