# Supplementary figures and images for: Chronic Cadmium Exposure Stimulates SDF-1 Expression in an ERα Dependent Manner
Source: PLoS One. 2013 Aug 28;8(8):e72639. doi: 10.1371/journal.pone.0072639 (PMC3755996; doi:10.1371/journal.pone.0072639)

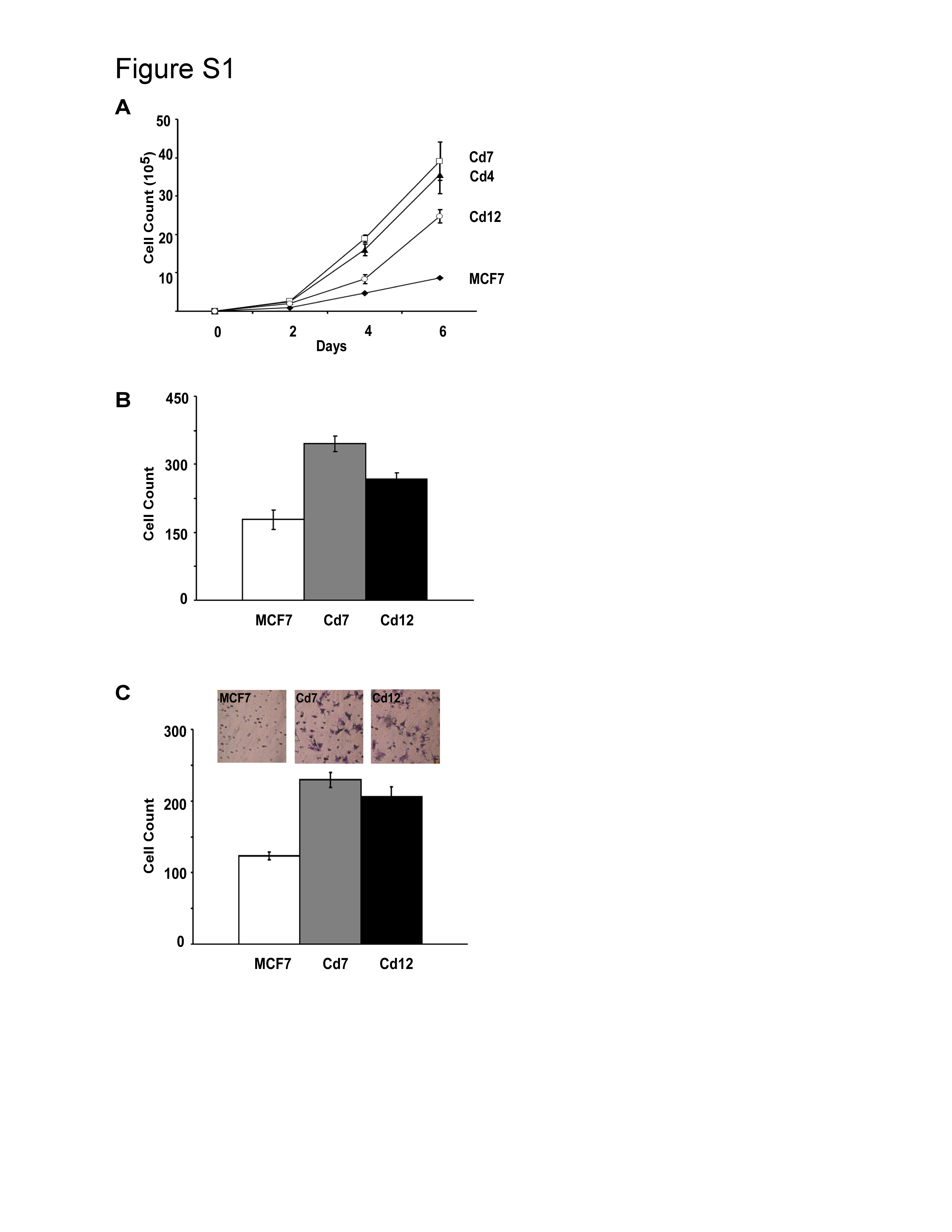

Supplement: Figure S1 — Cadmium-adapted cells display more aggressive cancer phenotypes: cell growth, migration and invasion. (A) MCF7-Cd clones (Cd4, Cd7, and Cd12) and parental MCF7 cells were plated in 6 well plates and growth was monitored 2, 4, and 6 days after plating by counting in triplicates with an automatic cell counter (P<0.01). Experimental results are representative of three independent experiments. (B) Modified-Boyden chamber assay was performed to measure the migration abilities of MCF-Cd cells. MCF7 (white bars), Cd7 (gray bars) or Cd12 (black bars) cells were seeded in upper chamber containing hormone-deprived media and allowed to migrate for16 hours to lower chamber containing media contain fetal bovine serum (FBS). Data is representative of 3 independent experiments done in triplicates (P<0.01). (C) An invasion assay was performed by seeding either MCF7 (white bars), Cd7 (gray bars) or Cd12 (black bars) cells in the upper chamber. Cells were allowed to invade through matrigel-coated membrane for 18 hours. Data is representative of 3 independent experiments done in triplicates (P<0.01). (TIF) [file pone.0072639.s001.tif]
